# Evaluating Reaction Videos of Young People Watching Edutainment Media (MTV Shuga): Qualitative Observational Study

**DOI:** 10.2196/55275

**Published:** 2025-01-31

**Authors:** Venetia Baker, Sarah Mulwa, David Khanyile, Georgia Arnold, Simon Cousens, Cherie Cawood, Isolde Birdthistle

**Affiliations:** 1 Faculty of Epidemiology and Population Health The London School of Hygiene and Tropical Medicine London United Kingdom; 2 Epicentre Health Research Durban South Africa; 3 GA-Agency London United Kingdom

**Keywords:** mass media, edutainment, adolescents, sexual health, HIV prevention, participatory research

## Abstract

**Background:**

Mass media campaigns, particularly edutainment, are critical in disseminating sexual health information to young people. However, there is limited understanding of the authentic viewing experience or how viewing contexts influence engagement with media campaigns. Reaction videos, a popular format in web-based culture in which users film themselves reacting to television shows, can be adapted as a research method for immediate and unfiltered insights into young people’s engagement with edutainment media.

**Objective:**

We explored how physical and social context influences young people’s engagement with *MTV Shuga*, a dramatic television series based on sexual health and relationships among individuals aged 15 to 25 years. We trialed reaction videos as a novel research method to investigate how young people in South Africa experience the show, including sexual health themes and messages, in their viewing environments.

**Methods:**

In Eastern Cape, in 2020, purposively selected participants aged 18 to 24 years of an evaluation study were invited to take part in further research to video record themselves watching *MTV Shuga* episodes with their *COVID-19 social bubble*. To guide the analysis of the visual and audio data, we created a framework to examine the physical setting, group composition, social dynamics, coinciding activities, and viewers’ spoken and unspoken reactions to the show. We identified patterns within and across groups to generate themes about the nature and role of viewing contexts. We also reflected on the utility of the method and analytical framework.

**Results:**

In total, 8 participants recorded themselves watching *MTV Shuga* episodes in family or friendship groups. Viewings occurred around a laptop in the home (living room or bedroom) and outside (garden or vehicle). In same-age groups, viewers appeared relaxed, engaging with the content through discussion, comments, empathy, and laughter. Intergenerational groups experienced discomfort, with older relatives’ presence causing embarrassment and younger siblings’ distractions interrupting the engagement. Scenes featuring physical intimacy prompted some viewers to hide their eyes or leave the room. While some would prefer watching *MTV Shuga* alone to avoid the self-consciousness experienced in group settings, others valued the social experience and the lively discussions it spurred. This illustrates varied preferences for consuming edutainment and the factors influencing these preferences.

**Conclusions:**

The use of reaction videos for research captured real-time verbal and nonverbal reactions, physical environments, and social dynamics that other methods cannot easily measure. They revealed how group composition, dynamics, settings, and storylines can maximize engagement with *MTV Shuga* to enhance HIV prevention education. The presence of parents and the camera may alter young people’s behavior, limiting the authenticity of their viewing experience. Still, reaction videos offer a unique opportunity to understand audience engagement with media interventions and promote participatory digital research with young people.

## Introduction

### Background

Edutainment has become a crucial tool for disseminating sexual health information, especially to young people [1-5]. Singhal and Rogers [6] define *edutainment* as the deliberate use of media to entertain and educate, aiming to enhance knowledge, foster positive attitudes, and change behavior. This approach has traditionally used various media formats, including radio, television, comic books, and music, to influence audience awareness, attitudes, and behaviors in their external environment to help create the necessary conditions for social change [6]. The Sabido methodology is an approach to edutainment using soap operas to promote positive social change by integrating educational messages into compelling narratives. It relies on social learning theory, which posits that people learn behaviors by observing others, especially when they see those behaviors rewarded [6]. The Sabido methodology uses this theory to create positive, negative, and transitional role models that viewers can relate to, aiming to shift behaviors by showing the consequences of these characters’ actions [6]. Audience engagement is key as the method emotionally invests viewers in the characters’ journeys, encouraging reflection and discussion. The discussions that edutainment generates are central to its ability to diffuse new ideas into the community, shifting not just individual behavior but also social norms [7].

Edutainment plays a crucial role in the field of communication for development across various development challenges, such as HIV and AIDS, sexual and reproductive health, domestic violence, and sustainable livelihoods [3,4,8-10]. In South Africa, which has the largest population of people living with HIV (UNAIDS [11]), both governmental and nongovernmental organizations have made significant investments in communication campaigns aimed at correcting misinformation and reducing HIV-related stigma [1,12]. Edutainment was key in correcting misinformation and reducing stigma during South Africa’s response to the HIV or AIDS epidemic [13-16].

Edutainment became a key component in raising awareness about HIV and AIDS in South Africa, particularly through the popular drama *Soul City* [10]. Created by the Soul City Institute, *Soul City* is a multilevel and culture-sensitive communication strategy centered on the television drama that sought change at the individual, community, and broader society levels to inspire social action and sociopolitical mobilization by incorporating community mobilization and action into the narrative [7,17]. It integrated HIV storylines and reached millions of viewers, showing positive effects on knowledge, attitudes, and behaviors related to HIV prevention [10].

Since *Soul City*’s success, HIV and sexual health edutainment have become a popular approach for reaching South African audiences, especially younger age groups. *MTV Shuga, Down South* (2017-present), which is the focus of this study, is a current edutainment series that aims to create storylines centered on HIV prevention, including pre-exposure prophylaxis (PrEP) and HIV self-testing, for young people, a critical population for addressing the high incidence and prevalence of HIV in South Africa [18]. *Down South* is part of the *MTV Shuga* mass media campaign created by the MTV Staying Alive Foundation, which aims to engage young people with positive sexual health messages using compelling and relatable storylines. *MTV Shuga* is part of a large media campaign and has reached millions of viewers worldwide through television and web streaming since 2009.

When evaluating health edutainment interventions, research has often relied heavily on surveys and structured interviews to determine effectiveness [4,5,10,19,20]. These types of studies have shown the mixed effects of edutainment on sexual health outcomes. While these studies can track measurable changes in attitudes, behaviors, and tangible outcomes—such as increased use of services—they do not explain why certain elements of edutainment work whereas others do not. To fully understand the impact of edutainment, other methods must be used to explore the nuanced ways in which audiences engage with the content in their everyday lives [8].

Many studies that use qualitative methods to evaluate edutainment dramas with HIV or sexual health storylines rely on self-reported, retrospective data obtained through qualitative interviews and focus groups [3,21-25]. These methods have proven valuable in providing deep insights into participants’ personal experiences, perceptions, and emotional responses to edutainment content. Such approaches are essential for understanding the mechanisms through which edutainment influences behavior, attitudes, and beliefs as they allow participants to articulate their experiences in their own words.

Moreover, the retrospective nature of the data collected in these studies can capture longer effects of edutainment [3,4,26,27]. This includes how the content continues to resonate with participants over time, influencing their decisions and actions. Focus groups facilitate discussions that elicit diverse perspectives and highlight shared experiences among participants, contributing to a more comprehensive understanding of edutainment’s effects [4,21,23,24,27-29]. However, several limitations are inherent in these qualitative methods. Social desirability bias, recall issues, and the power dynamics between participants and researchers are notable concerns that may compromise the reliability of the data. Furthermore, there is a tendency to sample participants who are already engaged with the content, potentially skewing the findings toward more favorable opinions [4,21,22,26].

While most studies have relied on retrospective researcher-led methods, alternative approaches such as participatory methods and observational evaluations have also been explored. Some studies have used participatory and observational methods such as storytelling and community screenings to evaluate audience engagement [26,28,29]. Observations have provided valuable insights into physical settings and collective experiences similar to those seen in theaters or live performances [30-32]. However, these insights may not fully reflect the diverse influences and mediating factors present in more intimate home environments, where most edutainment is consumed.

*MTV Shuga* has been evaluated using both quantitative and qualitative retrospective methods. The results of our own research using a web-based survey in the Eastern Cape, South Africa, have shown a positive association, consistent with a causal impact of *MTV Shuga “Down South”* (based in South Africa) on young people’s awareness and use of HIV prevention tools such as HIV self-screening and condoms and greater motivation among adolescents to use PrEP for HIV [4]. Evaluations of other *MTV Shuga* series produced in other contexts such as Nigeria and KwaZulu-Natal in South Africa also show positive effects of engaging with *MTV Shuga*, for example, on young people’s adoption of chlamydia and HIV testing, condoms, and contraception, as well as significant attitude shifts and increased disclosure of sexual violence among youth [33-35]. Qualitative evaluations using in-depth interviews and focus group discussions of *MTV Shuga*, including our own [21], have found that *MTV Shuga*’s relatable, unjudgmental content helps young people think through scenarios that relate to their real lives. They also describe how the discussions and debates inspired by *MTV Shuga* created a safe place to discuss sexual health, reducing stigma and increasing support [21,27]. These methods contributed valuable knowledge to *MTV Shuga*’s effectiveness and the mechanisms in which *MTV Shuga* works. Participants self-reported how social context can modulate the viewing experience. However, we lacked data on how those interactions happen in real time and the way in which people consume *MTV Shuga* in their everyday lives.

Edutainment evaluations rarely use participatory approaches to capture viewers’ natural, real-time interactions and reactions, including how mass media edutainment is experienced in its actual viewing environment. To gain a more comprehensive perspective, there is a need to capture the viewing experience and how the viewing context, including both social and physical elements, influences the effectiveness and impact of mass media edutainment. This holistic understanding can further enhance the effectiveness of sexual health messaging through edutainment.

### Objectives

In this paper, we explore the role of reaction videos as a method of evaluating an edutainment campaign. Our objectives for using reaction videos as a research method were to (1) create a remote approach to observation without the presence of a researcher in which young people could record their experiences of watching the *MTV Shuga* show in viewing groups and (2) understand how physical context and social dynamics influence young people’s experiences of watching *MTV Shuga* within their household or other social spaces, with family, relatives, and friends. We also sought to critique the successes and challenges of using self-filmed observation for research purposes to reflect on the utility of this technique more widely in qualitative investigations with young people.

We piloted and described the use of reaction videos self-filmed by viewers as part of a mixed methods evaluation of *MTV Shuga* in South Africa. Previously, young people’s real-life engagement with the *MTV Shuga* series has not been documented at the time or place of private viewing, for example, at home with family and friends. We hoped that self-filmed reaction videos would complement the self-reported data we collected [4,21,36] by revealing the physical and social context in which young people view sexual health media content and how that experience may influence the show’s effect.

Analyzing reaction videos can provide valuable insights into viewers’ real-time emotional and physical reactions within their contextual environment. These videos, which record people’s responses as they watch scenes from movies, television shows, music videos, or comedy sketches, have become a prominent feature in web-based and media culture. The rising popularity of reaction videos on YouTube, TikTok, and other social media platforms reflects a fascination with observing other people’s natural, unscripted social interactions. As with other media formats, reaction videos have the potential to be both entertaining and educational. They offer a literal lens with which we can observe the influence of media in real-time and real-life situations [37]. While viewers are aware of the recording and potentially alter their behavior consciously or unconsciously, reaction videos offer an inside perspective that cannot be easily captured otherwise and which can complement other data sources for research and evaluation.

There is an emerging body of literature on the nature and influence of reaction videos, primarily focusing on reaction videos on YouTube. Analysis of YouTube reaction videos reveals that they vary from amateur—unscripted, spontaneous, and organic—to more professional, scripted, and edited formats. While primarily aimed at entertainment, these videos also create narratives that guide viewers in interpreting content through the creators’ perspectives [38]. They help build communities and challenge dominant views, enabling viewers to share their own perspectives and influence how videos are consumed [38]. These videos foster a sense of community by uniting viewers worldwide to share and discuss in comment sections, with participatory elements such as content suggestions and creator interactions further strengthening these connections [39,40].

Adopting reaction videos as a research method presents valuable potential for data collection. Instead of analyzing existing reaction videos on the web, we asked participants to create their own reaction videos, which would not be posted on the web for entertainment but used exclusively for research purposes. This approach aimed to minimize the influence of performative, exaggerated reactions often seen in web-based content, where viewers may enhance their responses to build a fan base or generate income. We hoped that these reactions would be similar to what participants might naturally experience at home. This method can provide insights from individuals who are not web-based personalities who may be posting content for attention or commercial purposes, which can skew data toward extremes due to algorithmic pressures. By adopting this format, researchers can collect real-time responses and insights, offering a rich qualitative data source reflecting participants’ immediate reactions and emotions. This approach deepens data collection and provides a novel way to engage participants in the research process. The use of reaction videos as a research method can draw from forms of traditional data collection, including photovoice and observations. The participatory and visual principles of photovoice apply here as they empower participants to document and share their reactions and perspectives, fostering a more engaged and authentic form of data collection [41,42]. Similarly, the principles of observation can be used by capturing real-time interactions and spontaneous responses, providing rich, contextual insights. Video recordings can allow researchers to capture the participants’ physical environment, social dynamics, and other contextual factors without physically being there [43,44]. Reaction videos introduce an additional participatory dimension to video observations as the participants are responsible for self-recording, allowing them to decide what to capture.

## Methods

### Recruitment

We recruited participants from a web-based survey that was live on the web in South Africa from September 2020 to December 2020. The survey was part of an evaluation to understand the impact of the mass media campaign *MTV Shuga Down South season 2 (DS2)*, and detailed methods and results to date have been published previously [4,21,36]. In short, 3431 young people aged 15 to 24 years completed the web-based survey. Participants were recruited through targeted advertisements to individuals aged 15 to 24 years in Mthatha through social media platforms, primarily Facebook. The questionnaire was also promoted in collaboration with local schools, universities, and clinics through their Facebook pages and WhatsApp groups.

This study was conducted in Mthatha, Eastern Cape, which was selected due to the recent distribution of HIV self-testing kits and the availability of oral PrEP in the area, ensuring that any demand generated by the *MTV Shuga DS2* campaign could be met. In addition, the offline components of the *MTV Shuga DS2* campaign were active in this region, providing a local context for examining the campaign’s impact. Mthatha was also selected because of its relatively lower levels of HIV prevention research and testing compared to other provinces, making it a priority area for intervention. This decision was further supported by the South African Department of Health, which identified Mthatha as a strategic site for increasing HIV prevention efforts among youth.

Of those who reported ever having watched *MTV Shuga* *DS2* (N=238), 28.6% (n=68) within the study area of Mthatha in Eastern Cape opted in to further research to be conducted remotely due to COVID-19 restrictions at the time. Participants were first invited to individual interviews or focus groups, the findings of which are reported elsewhere [4,21], and participants of those activities were subsequently invited to create video recordings of themselves watching *MTV Shuga* *DS2* episodes. Initially, the eligibility criterion was that viewing groups should include a parent and their adolescent children (aged 15-19 years) to understand parent-child viewing dynamics. However, when it proved difficult to recruit adolescents willing to watch with their parents, eligibility was expanded to all 68 participants from Mthatha (aged 15-24 years) who opted in to further research to watch *MTV Shuga* *DS2* with any family members or housemates of their choice (within their COVID-19 “social bubble”). Of those, participants were called at random until 8 consented to and participated in the reaction videos.

The primary participant for each viewing group, recruited through focus groups and interviews, must have watched at least one episode of *MTV Shuga DS2*, but the other participants in the group did not require any previous exposure. We did not impose an age limit for this activity as the main participant was drawn from the evaluation study with individuals aged 15 to 24 years, within the target audience for *MTV Shuga*. As all participants responsible for organizing their viewing group had some familiarity with *MTV Shuga*, they were responsible for determining their viewing group and assessing the appropriateness of the situation.

A total of 8 groups was considered a suitable number to trial the method and analytical framework with a range of viewing groups. The decision to use a small sample size was justified by the study’s aim to gain in-depth insights into family dynamics and viewing behaviors. We predicted that we would reach saturation with 8 groups ensured. By focusing our resources on a small number of groups watching multiple episodes of *MTV Shuga*, we prioritized depth over breadth, allowing for meaningful interactions and a thorough understanding of each family’s viewing experience. Practical and budget constraints also influenced the sample size, ensuring a feasible research process.

### Ethical Considerations

This study was approved by the Biomedical Research Ethics Committee at the University of KwaZulu-Natal (reference BREC/00000477/2019), the London School of Hygiene and Tropical Medicine (reference 17996), and the World Health Organization (reference ERC.0003283). Ethics approvals covered all phases of the research, including participant recruitment and data collection and analysis. Participants, who were recruited through the web-based survey, provided web-based consent before joining the study. For participants aged <18 years, consent was obtained from parents or guardians, and this was done via email.

Participants who opted into the individual interviews or focus groups and were later invited to the reaction videos were provided with detailed information sheets and had the opportunity to discuss the study with data collectors over the phone. During these discussions, participants could ask questions to ensure that they fully understood the nature of their participation. Parents of participants aged <18 years were required to give consent via a form sent over email.

This study took several measures to ensure privacy and confidentiality. All participant data were anonymized. The reaction videos themselves were not disseminated outside of the research team. To maintain secrecy while illustrating moments from these videos, the research team commissioned a young South African artist, Lunga Jonas, to create graphic art representations of the footage. These artworks altered participants’ facial features to prevent identification while retaining the social and emotional context of the group viewings.

In recognition of the time that the participants contributed to the study, they were compensated with 100 rand (US $5.54) for their participation. This compensation was provided to ensure that participants were fairly acknowledged for their involvement without coercion or undue influence.

This study followed the COREQ (Consolidated Criteria for Reporting Qualitative Research) checklist to ensure the transparency and rigor of the research process, including participant selection and data collection and analysis procedures.

### Data Collection

Researchers conducted informed consent procedures via a WhatsApp phone call with the entire viewing group, specifically to confirm that they were aware of the reaction videos and their purpose and consented to being filmed. The survey participants signed a consent form via email on behalf of their viewing group. For participants aged <18 years, the written permission of parents and guardians was also confirmed via email. We asked participants to use a smartphone to video record their group watching *MTV Shuga DS2* episodes 5 and 6 on YouTube, both 22 minutes long. It was not required for participants to have seen episodes 1 to 4 before participating in the reaction videos. These episodes include storylines about PrEP, HIV self-screening, and gender-based violence. They also feature 2 scenes with physical intimacy that include kissing, removing clothes, and opening condoms. All participants were able to choose where, when, and with whom they would watch the episodes. The participants transferred their video recordings via WhatsApp to the research team, who deleted them from the research phone once downloaded to a password-protected private server.

In total, 4 researchers familiar with the study setting and fluent in the local language reviewed each reaction video and took notes, describing the setting, interactions, and significant verbal and nonverbal communication in ways agreed upon during researcher training to aid consistency. Nonverbal behavior was defined to encompass body movements and postures, along with temporal speech markers such as gaps and silences. Verbal behavior was characterized by spoken words, verbal expressions such as gasps and laughter, and the strength or emotive inflection in vocal expressions [45,46].

Following their review, researchers called the main participant of each viewing group via a WhatsApp “debrief” call to ask about the experience of watching *MTV Shuga DS2* with this group of people and whether they had any advice to improve the show. Participants’ responses were summarized and included in the notes. Research notes and debrief notes were recorded in English and translated from Xhosa into English as needed.

### Analysis

To analyze reaction videos, we created a framework to capture the social and physical context of people consuming edutainment in their everyday lives [8,19]. We designed a framework with the following components: *group composition*, *physical setting*, *coinciding activities*, *social dynamics*, and *reactions to the DS2 show and this research activity* (Textbox 1).

**Reaction video analysis framework.**

**Components**

1. Group composition: the characteristics of those involved in the viewing, including their age, gender, relationships with each other, and other relevant information provided by the participants.
2. Physical setting: this describes the physical space in which the participants viewed the *MTV Shuga DS2* episodes. It encompasses factors such as the location, the time of day, surrounding physical objects, lighting, and overall environment, as well as the mode or device through which they viewed *MTV Shuga DS2*.
3. Coinciding activities: this constitutes any concurrent activities happening during the viewing that might either enhance or distract from engagement with the show.
4. Social dynamics: this constitutes the interactions between participants during the viewings. It includes spoken dialogue or unspoken gestures and responses between members of the viewing group.
5. Reactions to the *MTV Shuga DS2* show: this encompasses any verbal or nonverbal reaction expressed by participants in response to the *MTV Shuga DS2* episodes and scenes they are watching. This includes body language, facial and bodily expressions (such as laughing or gasping), and their overall attitudes while watching (eg, bored, relaxed, awkward, or happy) as perceived by the researchers and from the debrief call with the main participant.
6. Reactions to the research activity: this refers to participants’ awareness of the camera, including speaking directly to the camera, their chosen positioning for the filming, and any statements they made in the debrief call about their experience of filming.

To apply our analytical framework to the coding process, we used the framework’s subheadings to code each video recording, observation note, and audio transcript. Short summaries were written for each video, organized by the framework’s subheadings. These summaries were then combined to identify codes within and across the videos. The lead analyst collaborated with the data collection researchers and a translator to discuss and interpret the findings, ensuring that they accurately reflected the participants’ experiences. These codes were synthesized into themes guided by the framework supported by specific data excerpts such as quotations from transcripts or descriptions of moments captured in the videos.

The researchers involved in conducting the follow-up call (Zulu and Xhosa, male and female, and aged <30 years) and the lead analyst (White, British woman, and aged 32 years) reflected on how their knowledge, attitudes, assumptions, and positions might affect the interpretation of the data. On the basis of these reflections, we determined that the initial analysis by the lead analyst should be verified with the other researchers and transcribers to ensure that the data were accurately summarized and interpreted.

## Results

### Group Composition

A total of 8 self-filmed reaction videos were received from 8 distinct viewing groups (Multimedia Appendix 1). Only 1 group (group 1) included a parent who filmed her 3 sons (aged 15-26 years) while watching the show together. Other groups included viewers ranging in age from 7 to 32 years. These groups, confined to “COVID-19 social bubbles,” primarily consisted of siblings and cousins. All groups included male individuals: 38% (3/8) included male individuals only and 62% (5/8) were mixed-gender groups. There were no exclusively female groups; all female viewers were accompanied by older male cousins or younger brothers who were not within the target age range for *MTV Shuga* (15-25 years).

### Physical Setting

As illustrated in Multimedia Appendix 1, the environments in which the groups convened to watch *MTV Shuga DS2* were diverse, with a blend of communal and secluded spaces. Use of laptops by all groups may have allowed for flexibility in where they chose to watch the show. Groups 5, 6, and 7 viewed the episodes in spacious living rooms with plush sofas and chairs, suggesting an evening setting or at least an ambiance with the curtains closed. Group 1, on the other hand, opted for a more structured viewing setup, congregating around a dining table during daylight hours. Groups 4 and 8 chose the privacy of bedrooms, where they huddled around laptops with siblings or cousins. These indoor gatherings were marked by an informal, relaxed ambience, with participants donning loungewear and pajamas and sporting bonnets, all contributing to the gatherings’ casual and comfortable essence.

In total, 67% (2/3) of the all-male groups, groups 2 and 3, opted for outdoor settings for their daytime viewings. Group 2 created a makeshift viewing area by arranging chairs around a crate situated beside an exterior wall. The 4 brothers in group 3 sought an enclosed environment, choosing to watch the episodes from the confines of a vehicle. Although they mostly had the windows and doors shut, they occasionally rolled them down to talk to passersby who approached the vehicle to greet the boys inside.

### Coinciding Activities

Communal activities often accompanied the viewing of the show, particularly in groups watching at home. For instance, group 6 and 7 participants combined viewing with eating a meal, whereas group 5 engaged in doing their hair. These activities illustrated the intimacy or familiarity of these groups; however, they also introduced potential distractions. Engaging in activities unrelated to the show led to conversations and actions that diverted attention away from the content being watched. As a result, participants might have missed crucial dialogue or scenes, highlighting a trade-off between enhancing the communal viewing experience and maintaining focus on the show’s narrative and messages.

Other distractions included people who were not involved coming to chat with the participants, which only happened in group 3, who met in the vehicle.

Phone use emerged as a significant distraction during the viewings. Although recording the viewings required smartphones, other phones were frequently present and easily accessible to participants. While most participants focused on the show, smartphones proved to be potential distractions. For instance, in group 2, a participant began scrolling through his phone for several minutes, diverting his attention away from the show. Similarly, in group 5, a viewer aged 13 years was less engaged with the show than her sister, who was aged 24 years; more absorbed in her phone; and visibly distracted by an ongoing text conversation, as indicated by her active notifications. Both the older sister and younger brother nudged her and told her to concentrate on the episodes. In addition, smartphones were sometimes deliberately used to “escape” the viewing. In group 7, during a scene that made him noticeably uncomfortable, one man turned to his phone to avoid watching the laptop screen.

Despite most groups experiencing some distractions, no group paused or rewound to avoid missing the show.

### Social Dynamics

#### Awkwardness

While most of the time viewers appeared relaxed around each other, intimate scenes showing couples kissing, opening condoms, or discussing PrEP created awkwardness across many groups. Viewers aged ≤15 years often responded to the intimate scenes by closing their eyes, covering their faces, or laughing nervously. In group 1, the mother often teased and joked with her sons about their visible discomfort during some scenes, playfully remarking that “it’s not that painful” when her children appeared awkward during an intimate scene about PrEP that ended with characters kissing and taking off clothing. Although the atmosphere appeared lighthearted, the boys, smiling shyly, laughing with flushed faces, shaking their head, and covering their eyes, were embarrassed watching these scenes with their mother. In group 6, the teenage girl looked up and smiled awkwardly, showing discomfort as she pushed her face onto a pillow to hide from the intimate scene. Her uncle remarked the following in the debrief:

It was awkward a little bit because my niece and I are not used to each other that much, so I was uncomfortable with some scenes.

In the debrief calls, groups 5, 7, and 1 agreed that watching with family was awkward, but overall, they said that they enjoyed the experience.

#### Dialogue

The reaction videos highlighted the social aspect of watching a show as a group, especially how it elicits reactions and facilitates discussion among viewers. In groups of young men, the show’s content related to HIV prevention, often intimate in nature, generated reactions or dialogue; for example, one participant in group 3 encouraged the characters on-screen to engage in sexual activity using the phrase “Shaya shaya [Hit Hit].” Participants explained how watching the show created an environment where they could discuss topics that came up on the show once the viewing was complete. Group 2 shared the following:

I love watching shows like these with my brothers because we discuss and agree and disagree on some issues afterwards, but it helps because we grow each other.

For group 3, the discussions were also a major component of enjoyment of the show; they stated that they “loved every moment of it because we discussed it afterwards and shed light, so it was very informative.” However, it was not universal that groups enjoyed watching the show in groups—mainly due to discomfort around intimate scenes and relationship dynamics with family members, as referenced in the aforementioned theme. As the group 6 uncle stated, he found it uncomfortable watching with his niece, but “I wouldn’t have a problem watching alone.”

#### Age Dynamics

This study revealed varying dynamics in viewing experiences across different age groups. Groups in which viewers were of a similar age, such as group 7, found the viewing experience agreeable and comfortable, noting the following:

It was okay [watching with family]. We are all adults.

Similarly, group 4 said the following:

We enjoyed it. It was fine because we are almost the same age group.

The presence of younger siblings aged 7 to 8 years in groups 5, 6, and 8 introduced unique dynamics. These younger viewers were notably active, often moving in and out of the room, yet engaged with the show by asking questions about the storyline, such as “What’s happening?” (group 8) or “Who is Lemo?” (group 5). These inquiries sometimes elicited impatience from their older siblings. For instance, during a scene in which a character discovers that his girlfriend has tested positive for HIV, the viewer aged 8 years in group 5 began narrating the event, only to be cut off by an older sister with a dismissive, “You don’t need to explain.” The video recording later captures the end of a discussion happening off camera, then shows a visibly upset younger brother remarking, “This is such a joke, this is supposed to be a family thing” before exiting the room.

In the group 8 recording, the younger brother playfully interacted with the camera, making faces and sticking out his tongue, prompting a reprimand from his older sister filled with a mix of discipline and humor:

If you do that one more time, I am going to make you rewatch this [laughs and pauses], but I’m serious.

These interactions show that, although younger viewers were included, their older siblings frequently guided or managed their behavior. It led to distractions for the older viewers and irritation of the younger ones.

### Reactions to the Videos

#### Entertainment Value

Overall, the participants seemed to enjoy watching the show, finding it engaging and fun. Many participants stated in the debrief that they enjoyed the music, and this was evident during the recordings, with some singing along and dancing in moments (groups 6 and 8) and recognizing famous actors on the show. Others enjoyed the other creative components integrated into the show; for instance, the older sister in group 5 hummed and snapped her fingers during a scene with a poetry slam.

#### Reactions to Storylines

##### Q and Dineo (Transactional Sex)

The storyline between Q and Dineo, which portrays a budding romantic relationship, elicited varied reactions from participants, particularly regarding the theme of transactional relationships. Dineo’s involvement in a transactional sexual relationship, where her school fees are paid by an older man (a “Blesser”), sparked comments on the motivations behind such relationships. In group 4, a viewer empathized saying, “Things we do for money” when Dineo explained to Q why she was sleeping with an older man.

Q’s initial response to Dineo’s situation was seen as understanding and supportive. The group 1 mother remarked, “That’s one understanding guy” as Q came to terms with Dineo’s arrangement with her Blesser.

However, Q’s attempts to win Dineo’s affection by spending money on her generated different reactions. In group 2, a male viewer aged 19 years noted Q’s financial expenditure, remarking, “u fit kanje,” which translates to “so much money.” This observation was echoed by other groups, with expressions of surprise and dismay watching Q withdraw his savings to spend on Dineo.

In group 3, participants expected physical intimacy in Q and Dineo’s relationship as a return for Q’s financial spending, encapsulated in the following comment—“Akamunike futhi (She must give it to him now)”—showcasing the perceived sexual obligation created by the financial exchange.

##### Reggie and Odirile (Same-Sex Relationship)

The responses of boys and men to scenes showing same-sex intimacy were characterized by notable discomfort. This was manifested by in-jokes and physically shielding their eyes during the scenes in which 2 male characters showed intimacy while discussing the use of PrEP. In groups 2 and 7, male viewers’ reactions to these scenes were marked by avoidance; a male individual from each group walked away when the 2 male characters started kissing. This strong aversion contrasted with a female viewer in group 7 who laughed at the boys’ uneasy reactions. In group 4, during a scene in which male characters Reggie and Odirile kissed, the group expressed their unease (shaking their head, covering their eyes, and groaning), with one stating, “They should move on to the next scene,” suggesting a collective discomfort or disinterest in engaging with same-sex romantic content. Young men’s reactions potentially led them to miss the scene and the educational content about PrEP and HIV prevention options.

##### Daniel and Ipeleng (HIV Self-Testing)

The HIV self-testing scenes in *MTV Shuga DS2* were designed to be educational and showed the characters Daniel and Ipeleng using a mouth swab for self-testing at home. This scene elicited a subdued reaction compared to scenes involving PrEP and intimacy. Only groups 7 and 5 visibly reacted to these moments. In group 5, someone off camera expressed disbelief saying, “i-swab? [a swab?],” showing that she was surprised that HIV could be screened for using an oral swab. Group 7 participants showed skepticism; one doubted the method’s reliability, preferring a blood test and saying, “Don’t trust this one; I want the blood one.” Another participant misconstrued the testing method’s implications, mistakenly suggesting, “If they swab, that means we can get HIV through kissing.”

### Reaction to the Research Method

The reaction videos were, on average, 29.38 (SD 9.3) minutes long. The duration of the 2 episodes together is 44 minutes. Some of the videos were sent in 1 long recording, whereas other groups sent multiple shorter videos.

The awareness or presence of the camera played different roles in each video. In group 1, the mother, holding her phone to film the observation, stood over her sons, using the camera to zoom in and capture their expressions. Although her voice was audible, she remained invisible on-screen. Her sons were very aware of the camera and often hid their faces, smiling shyly as she brought the camera close. In contrast, the young female participants who watched with their younger siblings (groups 5 and 8) positioned themselves as the central characters in the viewing, ensuring the camera captured their reactions. They appeared to be aware of the camera, sometimes glancing at it. For instance, the viewer aged 24 years in group 5 set up the camera and positioned herself within the frame. She took charge of the viewing, reprimanding her sister for checking her phone and not paying attention to the show. Her glances at the camera showed an awareness that the video had captured the distraction.

## Discussion

### Principal Findings

This study offers valuable insights into how physical and social contexts shape engagement with edutainment content such as *MTV Shuga*. Reaction videos captured nuanced dynamics within diverse viewing groups, including siblings, cousins, parents, and friends, revealing varying levels of comfort and discussion. Participants’ responses to themes such as same-sex intimacy, HIV prevention, and transactional sex were influenced by familial and peer relationships, as well as the settings in which they viewed the show—ranging from communal living rooms to private bedrooms and outdoor spaces. Concurrent activities such as eating, styling hair, and smartphone use further modulated the viewing experience. Our findings demonstrate that reaction videos effectively captured spontaneous engagement, providing unique insights into nonverbal cues, group dynamics, and emotional reactions often missed by retrospective methods. This real-time documentation deepened our understanding of how physical and social environments impact viewer engagement. While self-filmed observations proved valuable in capturing immediate reactions and discussions, challenges such as varying levels of camera awareness and engagement with the content could affect the authenticity of the viewing experience.

This approach leverages smartphone cameras to allow participants to show their physical and social context while enabling remote observation by researchers. This eliminates the need for a researcher’s physical presence, potentially reducing response bias as the presence of the in-person researcher will not influence social dynamics. While the absence of a researcher can diminish direct influence, the visible awareness of being filmed and the anticipation of sharing these recordings for research could shape participants’ behaviors.

However, while participants were aware of the camera, most did not directly engage with it. Unlike the highly entertainment-focused content on social media, the reaction videos in this research captured responses in natural viewing environments that appeared candid and unobtrusive [38,40]. The reactions recorded in this study likely reflect genuine, unguarded responses, similar to those occurring when no “audience” is watching the reaction. This contrasts with the more performative reactions often seen in content aimed at entertainment, persuasion, or monetization [38,39].

Granting participants the autonomy to decide their positioning in front of the camera and select the moments they wish to record transforms the research process into a participatory one. The dynamic of power and control shifts as participants handle the camera by self-focusing or navigating family interactions, such as older siblings monitoring their younger siblings’ interaction with the camera or parents capturing playful dominance over their children’s reactions. Participatory research is vital in adolescent research as it can examine power and voice while simultaneously being an empowering activity, building agency and giving voice to underrepresented or marginalized groups [47]. This shift in focus from researcher-controlled observation to participant-driven recordings highlights a key aspect of this study’s participatory methodology. Drawing from the principles of visual methodologies and participatory research rooted in the traditions of ethnography within anthropology and sociology [43,46], this method empowered participants to engage with the activity on their own terms.

The recordings enabled participants to document their reactions to *MTV Shuga* within the familiarity of their physical environment. Filming reactions in the comfort of their own homes or chosen environments, participants provided insights closely reflecting how they naturally might consume and engage with *MTV Shuga*. While open living rooms with communal activities created potential distractions, more intimate settings such as bedrooms allowed participants to engage deeply with the content. Outdoor spaces offered another setting for engagement as participants viewed in peer groups away from home. The distractions from young siblings and the disruptions of coinciding activities such as smartphone use or passersby could have impeded the educational and entertainment value of watching *MTV Shuga*.

While these various physical environments shaped the nature of participants’ engagement with the content, social dynamics within families also played a significant role in how the show was received. Even during the recruitment phase, participants resisted joining the qualitative reaction videos when we initially requested that adolescents (aged 15-19 years) watch the show with their parents. While we initially hypothesized that *MTV Shuga* could have a “family influence” by encouraging communication between parents and adolescents, we have since observed resistance to this in other research activities, including in-depth interviews and focus group discussions, in which adolescents cite discomfort in viewing sexually explicit content with parents for fear of awkwardness and judgment [21,27,36]. Some parents shared an interest in viewing and learning from the show, but encouraging “family” or intergenerational viewings can depend on the cultural context, for example, whether social norms about adolescents or sex can be challenged safely.

Participants noted that watching with peers of similar ages made the experience more engaging, sparking discussions and learning opportunities. The groups that reported engaging in postviewing discussions were mainly young men who viewed the show outside their homes. As observed in our previous study, creating youth-friendly spaces for media consumption could further enhance engagement with sexual health content [21].

Reaction videos capture the spontaneity of viewers’ real-time responses, providing insights into their immediate emotional reactions to the show’s content. Follow-up questions with a researcher further enrich these data, allowing participants to reflect on and articulate their experiences. This approach offers a dual layer of insight—combining initial reactions with more reflective postviewing feedback.

In particular, real-time reactions among viewing groups with mixed ages revealed that visual intimacy scenes in *MTV Shuga*, such as characters kissing, undressing, or reaching for condoms, caused discomfort for many participants. This led to distractions and awkwardness as participants covered their faces, walked out of the room, or picked up their phones often during critical and short HIV prevention scenes. It is not clear whether these reactions were due to the presence of others in the viewing group or the presence of the camera (spurred by social expectations) and may not have happened in the absence of either.

Despite the embarrassment caused by visual intimacy, participants approached discussions about sexual topics with more comfort when not accompanied by intimate scenes. For instance, several groups engaged in animated discussions about Q and Dineo’s relationship and transactional sex. Groups of young men, for example, were content watching scenes of Reggie and Odirile discussing sexual health but reacted negatively when the couple kissed on-screen.

These findings suggest that *MTV Shuga* may be more effective in facilitating conversations around sexual health and relationships if it reduces the intimate scenes that make viewing uncomfortable. However, the show must balance its aim of creating destigmatized representative content (eg, to help normalize kissing between a same-sex couple) with creating content that is comfortable for its audience [48]. In addition, in some cases, generating a strong or discomfiting reaction can challenge viewers to think and learn and leave a more lasting impression as long as it is not harmful or gratuitous. Reducing intimate scenes could dilute the drama and sexual appeal that make the show compelling, potentially diminishing its appeal to younger viewers.

Beyond these broader social dynamics, participants’ responses to specific content within *MTV Shuga* revealed key insights into the reception of public health messages. For example, the HIV screening scene, particularly the depiction of a swab for self-screening, generated limited dialogue and, concerningly, misconceptions about HIV transmission through saliva. Similar findings were documented in our earlier research [4]. To prevent misunderstandings and concerns among viewers, future series can embed clearer explanations of how oral swabs check for HIV antibodies in saliva and do not detect the HIV virus itself given the absence of real-time clarification from health professionals in media interventions.

Alongside responses to HIV prevention content, participants’ reactions to the show’s musical elements revealed how music shaped their engagement with the show. Reaction videos captured the immediate somatic responses of viewers to the music featured in *MTV Shuga* as they sang, danced, and snapped their fingers to the rhythm. Music holds a significant place in South African edutainment, particularly in programs that combine performance theatre and music to promote public health and HIV awareness [30-32]. These artistic methods leverage music’s emotional and rhythmic power to create a sense of interconnectedness and euphoria among audiences, making them more receptive to new ideas and behaviors [49].

Young people find the integration of music into health interventions both engaging and refreshing, which is crucial given that they are often overwhelmed and fatigued by the constant stream of HIV messaging and promotions. This saturation can lead to disengagement, making innovative approaches such as music-based edutainment particularly valuable in capturing their attention and encouraging meaningful engagement [32].

### Limitations and Adaptations

The findings of this study, involving a small sample of 8 reaction videos from a specific region of South Africa, may not be generalizable to other social and cultural contexts. The demographics of the participants do not represent *MTV Shuga* viewers in South Africa in general as no groups were female only. Female individuals constitute the largest viewing group of the MTV series [22]. Recruitment challenges, especially in engaging parents, impacted this study’s original design, constraining insights into parent-child dynamics during *MTV Shuga* viewings.

This study was conducted during the COVID-19 pandemic, likely impacting participants’ spaces and viewing habits. As a result, the viewing environment may not reflect the participants’ natural choices. COVID-19 restrictions also limited participants’ ability to select their preferred viewing partners, potentially affecting the authenticity of group composition and individual responses. Their reactions may have been influenced by peers’ or family members’ collective atmosphere and expectations.

Not all participants adhered to instructions to ignore the camera, indicating that the instructions were not clear to all and may have caused variation in the participants’ reactions to the activity. Nonetheless, their interactions with the camera provided valuable insights into social dynamics.

The success of this method hinges on participants’ ease with technology and access to the necessary resources, such as the internet, smartphones, and a laptop, further restricting who would be able to participate in this study. Challenges arising from the reaction video method included participants using personal devices for filming, impacting the quality of audio and visuals in the recordings. The videos’ narrow view makes it challenging to understand dynamics outside the camera’s frame within the room or other spaces.

A final limitation of the reaction video method is that it primarily captures only what is externally visible, audible, or physical. It fails to delve into the internal processes, such as thoughts and emotions, that are crucial for understanding the deeper cognitive and emotional responses of participants. As a result, a significant layer of participant experience remains unexplored.

The reaction videos provided valuable insights into both the social context and content engagement, and these findings also point to opportunities for expanding the approach in future studies. By adopting a more interactive research design, reaction videos could capture different types of data, offering deeper insights into participants’ thoughts and experiences. Researchers could capture more verbal data and gain insights into inner thoughts by asking participants to speak directly into the camera and express their thoughts more overtly. This method captures thoughts and feelings lost in a less interactive observational approach. Reaction videos can also be adapted to solo reactions, which could yield insights into more personalized viewpoints, again akin to those shared on social media platforms. As noted by some participants, some would be more comfortable viewing *MTV Shuga* alone.

### Conclusions

This study’s exploration of viewer engagement with *MTV Shuga* through reaction videos illuminated the nuanced impact of physical and social environments on the consumption and influence of edutainment. By capturing real-time reactions and discussions among various viewing groups in their chosen settings, this research method offered valuable insights into the immediate emotional responses and subsequent reflections on themes of intimacy, HIV prevention, and transactional sex. In addition, the innovative use of these reaction videos represents a significant opportunity for adolescent health research. This participatory approach respects young people’s autonomy and perspectives and provides deeper insights into their interactions with health-related content. Furthermore, it showed reaction videos to be a valuable method to capture dimensions of context, the physical and social context in which edutainment is consumed, which has not been documented using other evaluation methods.

This study acknowledges the inherent limitations in capturing the authentic viewer dynamics due to the self-selective nature of the viewing groups, the challenges posed by the COVID-19 context, and the observer effect of the camera. The findings suggest that, while reaction videos can significantly enrich our understanding of audience reception, careful consideration must be given to the potential influences on participant behavior and the representativeness of the data collected.

## Appendix

Multimedia Appendix 1 Illustrations and descriptions of the viewing groups partaking in the self-filmed reaction videos.

## Abbreviations

COREQ: Consolidated Criteria for Reporting Qualitative Research
DS2: Down South season 2
PrEP: pre-exposure prophylaxis

## Data Availability

The datasets generated during and analyzed during this study are not publicly available due to the identifiable nature of the data but nonidentifiable data such as transcripts and summaries are available from the corresponding author on reasonable request.
