# Supplementary figures and images for: Evaluating Reaction Videos of Young People Watching Edutainment Media (MTV Shuga): Qualitative Observational Study
Source: JMIR Form Res. 2025 Jan 31;9:e55275. doi: 10.2196/55275 (PMC11829175; doi:10.2196/55275)

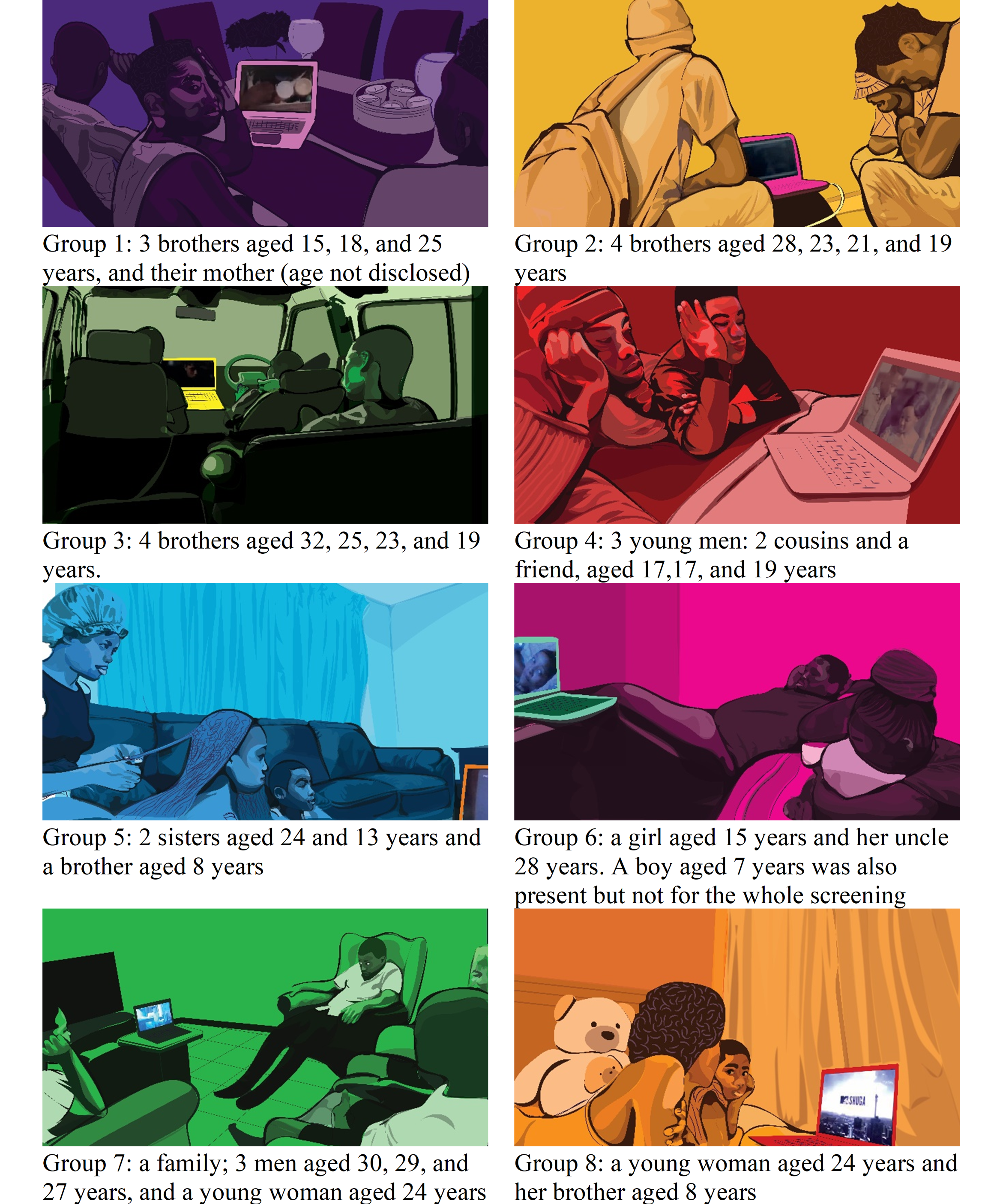

Supplement: Multimedia Appendix 1 [file formative_v9i1e55275_app1.png]
